# SGLT2 inhibitors for the prevention and treatment of heart failure: A scientific statement of the HFA and the HFAI

**DOI:** 10.1002/ehf2.15408

**Published:** 2025-09-19

**Authors:** Marco Metra, Daniela Tomasoni, Marianna Adamo, Offer Amir, Stefan D. Anker, Antoni Bayes‐Genis, Michael Boehm, Javed Butler, Ovidiu Chioncel, Gerasimos Filippatos, Finn Gustafsson, Ewa A. Jankowska, Juan Carlos Kaski, Brenda Moura, Mark C. Petrie, Piotr Ponikowski, Amina Rakisheva, Arsen Ristic, Francois Roubille, Gianluigi Savarese, Petar Seferovic, Peter van der Meer, Maurizio Volterrani, Andrew J. Coats, Vijay K. Chopra, Giuseppe Rosano

**Affiliations:** ^1^ Cardiology. ASST Spedali Civili di Brescia and Department of Medical and Surgical Specialties, Radiological Sciences, and Public Health University of Brescia Brescia Italy; ^2^ Department of Clinical Science and Education Södersjukhuset, Karolinska Institutet Stockholm Sweden; ^3^ Department of Cardiology, Hadassah Medical Center, Faculty of Medicine Hebrew University of Jerusalem Jerusalem Israel; ^4^ Department of Cardiology (CVK) of German Heart Center Charité German Centre for Cardiovascular Research (DZHK) partner site Berlin, Charité—Universitätsmedizin Berlin Berlin Germany; ^5^ Heart Institute Hospital Universitari Germans Trias i Pujol, Badalona, CIBERCV Barcelona Spain; ^6^ Klinik für Innere Medizin III, Universitätsklinikum des Saarlandes Saarland University Homburg Germany; ^7^ Baylor Scott & White Research Institute Dallas Texas USA; ^8^ University of Mississippi Jackson Mississippi USA; ^9^ Emergency Institute for Cardiovascular Diseases ‘Prof. C.C. Iliescu’ University of Medicine Carol Davila Bucharest Romania; ^10^ School of Medicine National and Kapodistrian University of Athens Athens Greece; ^11^ Department of Cardiology, The Heart Centre Rigshospitalet, University of Copenhagen Copenhagen Denmark; ^12^ Institute of Heart Diseases Wrocław Medical University Wrocław Poland; ^13^ Molecular and Clinical Sciences Research Institute St George's University of London London UK; ^14^ Centro de Investigação em Tecnologias e Serviços de Saúde Porto Portugal; ^15^ Serviço de Cardiologia Hospital das Forças Armadas—Pólo do Porto Porto Portugal; ^16^ School of Cardiovascular and Metabolic Health University of Glasgow Glasgow UK; ^17^ Department of Cardiology Wrocław Medical University Wrocław Poland; ^18^ Department of Cardiology Scientific Institution of Cardiology and Internal Diseases Almaty Kazakhstan; ^19^ School of Medicine University of Belgrade Belgrade Serbia; ^20^ PhyMedExp Université de Montpellier, INSERM, CNRS, Cardiology Department, CHU de Montpellier Montpellier France; ^21^ Serbian Academy of Sciences and Arts Belgrade Serbia; ^22^ Department of Cardiology University Medical Center Groningen, University of Groningen Groningen The Netherlands; ^23^ Department of Medical Sciences, Centre for Clinical and Basic Research IRCCS San Raffaele Pisana Rome Italy; ^24^ Heart Research Institute Newtown New South Wales Australia; ^25^ Department of Cardiology Medanta Gurgaon Haryana India; ^26^ Department of Human Sciences and Promotion of Quality of Life San Raffaele Open University of Rome Rome Italy; ^27^ IRCCS San Raffaele Rome Italy

**Keywords:** GDMT, heart failure, prevention, SGLT2 inhibitors, treatment

## Abstract

In the 2021 European Society of Cardiology (ESC) heart failure (HF) guidelines, sodium–glucose cotransporter 2 (SGLT2) inhibitors were recommended for the prevention of HF in patients with type 2 diabetes mellitus (T2DM) and for the treatment of HF with reduced ejection fraction (HFrEF). Further trials showed efficacy of empagliflozin and dapagliflozin in patients with HF with preserved ejection fraction (HFpEF). These results prompted a broadened recommendation for the SGLT2 inhibitors dapagliflozin or empagliflozin across the whole left ventricular ejection fraction (LVEF) spectrum in the 2023 Focused Update of the ESC HF guidelines and in other international guidelines. In SOLOIST‐WHF and EMPULSE, sotagliflozin (enrolling only patients with T2DM) and empagliflozin, respectively, were beneficial when initiated at the end or soon after an episode of decompensated HF. Based on these results and on the early appearance of their beneficial effects, the administration of SGLT2 inhibitors should start early in patients hospitalized for acute HF. Analyses after study drug withdrawal in randomized clinical trials have shown that their benefits may decline rapidly after discontinuation, and thus, persistence of treatment is advised. In EMPACT‐MI, empagliflozin did not reduce the primary outcome of cardiovascular (CV) death/HF hospitalization but reduced first/recurrent HF hospitalizations. Potential benefits of SGLT2 inhibitors in further specific conditions (i.e., cardiac amyloidosis, grown‐up congenital heart disease and paediatric patients with HF) have been reported in observational studies but need confirmation from prospective trials. This scientific statement summarizes current evidence regarding the effects of SGLT2 inhibitors for the prevention and treatment of HF.

## Introduction

Heart failure (HF) entails a poor prognosis, and sodium–glucose cotransporter (SGLT) type 2 (SGLT2) inhibitors (SGLT2is) are now among the pillars for its prevention and treatment. Dapagliflozin, canagliflozin and empagliflozin demonstrated a reduced occurrence of HF hospitalizations in patients with type 2 diabetes mellitus (T2DM), thereby establishing their pivotal role for HF prevention, leading to their recommendation for the prevention of HF in patients with T2DM at high risk or with cardiovascular (CV) disease.^1^ Following the evidence of the preventive effect in patients with T2DM with and without underlying CV disease, dapagliflozin, empagliflozin and sotagliflozin have then been assessed in patients with HF irrespective of the presence of diabetes. Thereafter, based on the results of DAPA‐HF (Dapagliflozin And Prevention of Adverse outcome in Heart Failure) and EMPEROR‐Reduced (Empagliflozin Outcome Trial in Patients with Chronic Heart Failure and a Reduced Ejection Fraction), the SGLT2is dapagliflozin and empagliflozin were recommended in both the 2021 European Society of Cardiology (ESC) and 2022 American College of Cardiology (ACC)/American Heart Association (AHA)/Heart Failure Society of America (HFSA) HF guidelines as foundational therapy for patients with HF with reduced ejection fraction (HFrEF) to reduce the risk of HF hospitalization and CV death, and sotagliflozin has been indicated for the treatment of diabetic patients with HF.^2^, ^3^, ^4^, ^5^, ^6^

Additional randomized controlled clinical trials with SGLT2is were accomplished after the 2021 ESC guidelines.^5^ EMPEROR‐Preserved (Empagliflozin Outcome Trial in Patients with Chronic Heart Failure with Preserved Ejection Fraction) and DELIVER (Dapagliflozin Evaluation to Improve the Lives of Patients with Preserved Ejection Fraction Heart Failure) met their primary composite endpoint in patients with HF and left ventricular (LV) ejection fraction (LVEF) >40%.^7^, ^8^ Their implications for clinical practice were deemed as so significant to prompt an update of the ESC guidelines 2 years after the former full‐length version.^5^, ^9^

Furthermore, new recommendations for the prevention of HF in patients with chronic kidney disease (CKD) were prompted by the results of the EMPA‐KIDNEY (Study of Heart and Kidney Protection with Empagliflozin) trial and a large meta‐analysis including previous trials.^10^, ^11^, ^12^, ^13^, ^14^ Treatment options for HF with preserved ejection fraction (HFpEF), CKD and metabolic conditions (e.g., obesity and diabetes) have expanded in recent years, raising practical questions regarding combination therapies.^15^ Additional trials with SGLT2is for the prevention of HF in patients after myocardial infarction (MI), as well as for the treatment of patients hospitalized due to acute HF, have been published to date.

**Figure 1:**
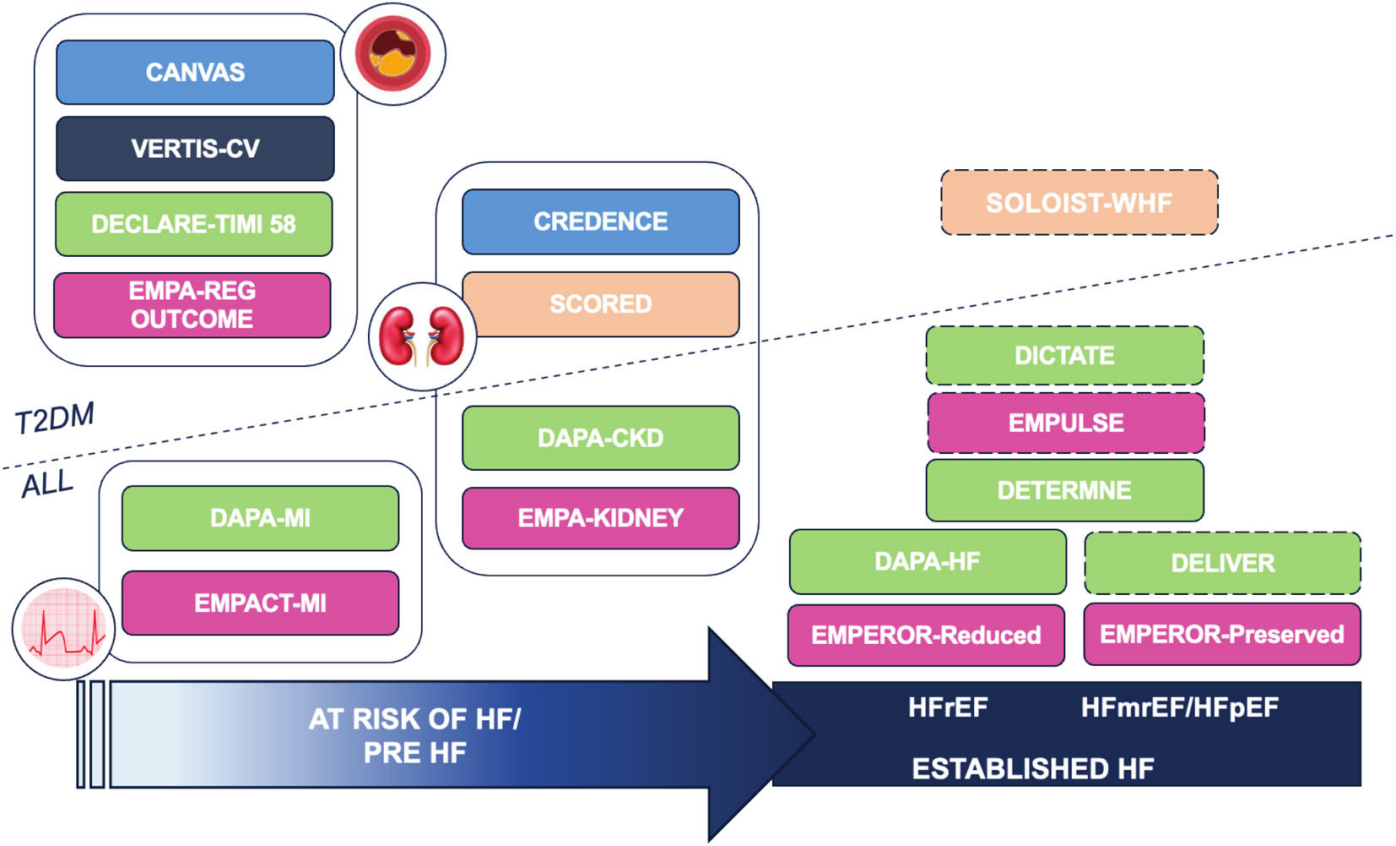
Trials with sodium–glucose cotransporter 2 inhibitors for the prevention and treatment of heart failure (HF). Dotted lines indicate trials including also patients during HF hospitalizations or with a recent episode of worsening HF. HFmrEF, HF with mildly reduced ejection fraction; HFpEF, HF with preserved ejection fraction; HFrEF, HF with reduced ejection fraction; T2DM, type 2 diabetes mellitus.

The aim of this scientific statement by the Heart Failure Association (HFA) of the ESC in collaboration with the Heart Failure Association of India (HFAI) is to summarize current evidence with SGLT2is issued after the publication of the 2021 ESC HF guidelines and having an impact on clinical practice (Figure 1).

## Prevention of HF

### T2DM and established atherosclerotic CV disease

The serendipity for SGLT2is and HF originated from the EMPA‐REG OUTCOME trial (Empagliflozin Cardiovascular Outcome Event Trial in Type 2 Diabetes Mellitus Patients), which demonstrated a 35% relative risk reduction in hospitalization for HF in patients with T2DM and established atherosclerotic CV disease who were treated with empagliflozin. Additional SGLT2is (canagliflozin, dapagliflozin and ertugliflozin) and the dual SGLT1 inhibitor/SGLT2i sotagliflozin were studied in large outcome trials in patients with T2DM and atherosclerotic CV disease or at high risk of CV events.^10^, ^12^, ^16^, ^17^, ^18^ Although the results of the trials differed with respect of major CV events and CV deaths, with significant reduction in CV death only in EMPA‐REG OUTCOME trial, a reduction in HF hospitalizations was shown in all trials with no heterogeneity across them.^14^, ^19^, ^20^, ^21^, ^22^, ^23^ In addition to meta‐analyses, a target trial emulation showed no differences between dapagliflozin and empagliflozin in 6 year CV outcomes in adults with T2DM with or without coexisting atherosclerotic CV disease or HF.^24^

The number of HF hospitalizations in these trials was rather small, with, hence, a small absolute risk reduction (ARR). For instance, in EMPA‐REG OUTCOME, HF hospitalizations were reduced by 35% [hazard ratio (HR) 0.65; 95% confidence interval (CI) 0.50–0.85] based on a reduction to 0.94 HF hospitalizations per 100 patient‐years on empagliflozin versus 1.45 HF hospitalizations per 100 patient‐years on placebo, with an ARR of 0.51 HF hospitalizations per 100 patient‐years. Even smaller values were reached in other trials, though with no heterogeneity across them.^17^, ^18^, ^19^, ^21^ Biomarkers, namely, N‐terminal pro‐brain natriuretic peptide (NT‐proBNP) and high‐sensitivity troponin T (hs‐TnT), may help select the patients at higher risk in whom a larger benefit from SGLT2i administration may be expected.^25^

Based on the results of major randomized controlled trials (RCTs) and meta‐analyses, SGLT2is were recommended for the prevention of HF in patients with T2DM and atherosclerotic CV disease or at high CV risk in the 2016 ESC HF guidelines.^1^ This indication was confirmed in the 2021 ESC HF guidelines.^1^, ^5^, ^16^, ^17^, ^18^, ^26^ In the 2023 ESC guidelines for the management of CV disease in patients with diabetes, SGLT2is (empagliflozin, canagliflozin, dapagliflozin, ertugliflozin or sotagliflozin) were recommended in patients with T2DM with CV risk factors or established atherosclerotic CV disease to reduce the risk of HF hospitalization.^27^

### Differences between SGLT inhibitors

There are major differences in the relative inhibition of SGLT1 and SGLT2 among the SGLT inhibitors (e.g., sotagliflozin has a 20× selectivity for SGLT2, compared with SGLT1, in comparison with a 2500× selectivity and 1200× selectivity for empagliflozin and dapagliflozin, respectively), and this may explain different benefits on major adverse cardiovascular event (MACE).^28^ SGLT1 is a low‐capacity, high‐affinity glucose transporter expressed in the late renal proximal tubule, where it is responsible for absorbing around 3% of urinary glucose in normal individuals. It is also expressed in the capillaries of the heart, brain and skeletal muscle and, mainly, in the brush border of the small intestine, where its inhibition results in an alteration in the intestinal microbiome that leads to an increase in the secretion of the incretin glucagon‐like peptide‐1 (GLP‐1) and a decrease in glucose‐dependent insulinotropic polypeptide (GIP). GLP‐1 increase might be responsible for a greater reduction in the incidence of stroke and, to a lesser extent, MI observed with the SGLT1 inhibitor/SGLT2i sotagliflozin compared with SGLT2 inhibition alone, through a decrease in platelet activation and an increase in atherosclerotic plaque stability.^28^, ^29^

### CKD

CKD is another major risk factor for the development of HF.^30^, ^31^ The main results of trials testing SGLT2is in patients with CKD, with or without diabetes, are summarized in Table 1.^10^, ^11^, ^12^, ^13^, ^32^, ^33^, ^34^, ^35^, ^36^ Meta‐analyses of SGLT2i trials issued after the 2021 ESC HF guidelines showed the efficacy of SGLT2is to prevent new HF events in patients with CKD and diabetes, but not in patients with CKD and no diabetes, likely for the small number of events.^14^, ^22^ Based on these results, SGLT2is were recommended in patients with CKD and T2DM to reduce the risk of HF hospitalization and CV death in the 2023 HF guidelines update and to reduce the risk of CV death and kidney failure in the 2023 ESC guidelines for the management of CV disease in patients with T2DM.^9^, ^27^

**Table 1:** Major trials demonstrating CV benefits of SGLT2is in patients with CKD.

Study name and year	Drug comparisons	Number of pts	Main eligibility criteria	Primary outcome	Effect of treatment on primary outcome HR; 95% CI	HF‐related event reduction HR; 95% CI	Events per 100 patient‐years (SGLT2i vs. placebo)
CREDENCE, 2019^10^	Canagliflozin (100 mg once daily) or placebo	4401^a^	eGFR 30–90 mL/min/1.73 m^2^ and albuminuria (uACR 300–5000 mg/g) T2DM Stable dose of an ACEi/ARB	Composite of ESKD^b^, a doubling of the serum creatinine level, or death from renal or CV causes	0.70; 0.59–0.82	HFH, 0.61; 0.40–0.80	1.6 vs. 2.5 (HFH)
DAPA‐CKD, 2020^11^, ^32^	Dapagliflozin (10 mg once daily) or placebo	4304	eGFR 25–75 mL/min/1.73 m^2^ and uACR 200–5000 mg/g With or without T2DM Stable dose of ACEi/ARB for ≥4 weeks	Composite of a sustained ≥50% decline in eGFR, ESKD^b^, or death from renal or CV causes	0.61; 0.51–0.72	HFH, 0.51; 0.34–0.76	0.8 vs. 1.6 (HFH)
SCORED, 2021^12^	Sotagliflozin (200–400 mg once daily) or placebo	10584	eGFR 25–60 mL/min/1.73 m^2^ T2DM At risk for CV disease	Total number of deaths from CV causes, HFH and urgent visits for HF^c^	0.74; 0.63–0.88	Total number of HFHs and urgent HF visits (first secondary endpoint), 0.67; 0.55–0.82	3.5 vs. 5.1 (HFH and urgent visits for HF)
EMPA‐KIDNEY, 2023^13^	Empagliflozin (10 mg once daily) or placebo	6609^a^	eGFR 20–44 mL/min/1.73 m^2^ or eGFR 45–89 mL/min/1.73 m^2^ with a uACR ≥ 200 mg/g With or without T2DM Treated with RAS inhibitor, unless not tolerated	Composite of kidney disease progression (ESKD^d^, renal death, sustained decline in eGFR to <10 mL/min/1.73 m^2^ or a ≥40% decline from randomization) or CV death	0.72; 0.64–0.82	Composite outcome of HFH or CV death, 0.84; 0.67–1.07	2.0 vs. 2.4 (HFH or CV death)

Abbreviations: ACEi, angiotensin‐converting enzyme inhibitor; ARB, angiotensin receptor blocker; CI, confidence interval; CKD, chronic kidney disease; CV, cardiovascular; eGFR, estimated glomerular filtration rate; ESKD, end‐stage kidney disease; HF, heart failure; HFH, heart failure hospitalization; HR, hazard ratio; MACE, major adverse cardiovascular event; pts, patients; RAS, renin–angiotensin system; SGLT2i, sodium–glucose cotransporter 2 inhibitor; T2DM, type 2 diabetes mellitus; uACR, urinary albumin‐to‐creatinine ratio.

^a^ The trial was stopped early after a planned interim analysis based on the recommendation of the data and safety monitoring committee due to efficacy.

^b^ ESKD is defined as dialysis, transplantation or a sustained eGFR of <15 mL/min/1.73 m^2^.

^c^ The original coprimary endpoints, assessed in time‐to‐event analyses, were the first occurrence of a MACE (defined as death from cardiovascular causes, nonfatal myocardial infarction or nonfatal stroke) and the first occurrence of death from cardiovascular causes or hospitalization for heart failure. Because of the early closing of the trial and the fewer‐than‐planned number of events, with the investigators and sponsor unaware of the trial group assignments and without endpoint information from an interim analysis, the primary endpoint was changed on 21 August 2020.

^d^ ESKD is defined as the initiation of maintenance dialysis or receipt of a kidney transplant.

No data are available for patients with severe CKD, as patients with an estimated glomerular filtration rate (eGFR) <20, <25 or <30 mL/min/m^2^ were excluded from trials.^11^, ^12^, ^13^, ^16^ Thus, initiation of SGLT2is cannot be advised in patients with a severe impairment in kidney function.^5^, ^6^

Of note, despite significant *P* values, the ARRs for HF events were small in the trials in patients with CKD because of their relatively small number. ARRs ranged from 0.25 to 1.04 per 100 patient‐years in the trials in patients with diabetes and from 0.80 to 1.39 per 100 patient‐years in the CKD trials. Hence, the number needed to treat (NNT) ranged from 95 to 400 patients in the diabetes trials and from 72 to 125 patients in the CKD trials.^37^ Nevertheless, relative risk reductions remained meaningful (12%–34% and 29%–31%, respectively) and consistent across different trials.^22^, ^37^

## Patients at risk after an acute MI

The effects of early SGLT2i initiation after an acute MI were investigated in two major trials, DAPA‐MI (Dapagliflozin in patients with myocardial infarction)^38^ and EMPACT‐MI (Empagliflozin on Hospitalization for Heart Failure and Mortality in Patients with Acute Myocardial Infarction).^39^ They were both neutral on their primary endpoints (CV death or HF hospitalization and all‐cause death or HF hospitalization, respectively). Of note, the residual risk of the enrolled patients was low. EMPACT‐MI selected patients at risk for HF, defined by either evidence of a newly developed LVEF < 45% or signs or symptoms of congestion requiring treatment during the index hospitalization (or both) and at least one additional enrichment factor. Additional risk factors were broad (e.g., age ≥65 years, severe LV dysfunction, and also three‐vessel coronary artery disease or peripheral artery disease). Examining the individual components of the primary endpoint, the risk of first HF hospitalization (HR 0.77; 95% CI 0.60–0.98), but not of all‐cause death (HR 0.96; 95% CI 0.78–1.19), was significantly reduced with empagliflozin as compared with placebo.^40^, ^41^ Due to the small number of events, the effect on HF hospitalization was, however, small in absolute terms, with an HF hospitalization rate of 5.9 events per 100 patient‐years on empagliflozin versus 6.6 events per 100 patient‐years on placebo.^41^ EMPRESS‐MI, using cardiac magnetic resonance imaging, failed to show effects of early treatment with empagliflozin on cardiac volumes or LVEF in patients with post‐MI LV systolic dysfunction receiving contemporary standard of care.^42^

## Treatment of HF

### HFrEF

SGLT2is are one of the four foundational drugs for HFrEF, along with angiotensin receptor–neprilysin inhibitors (ARNIs)/angiotensin‐converting enzyme inhibitors (ACEis), beta‐blockers and mineralocorticoid receptor antagonists (MRAs).^4^, ^5^, ^6^ In the DAPA‐HF and EMPEROR‐Reduced trials, treatment with dapagliflozin and empagliflozin, compared with placebo, in addition to optimal medical therapy, reduced the risk of major HF outcomes by 25%–26% in ambulatory patients with HFrEF (Table 2).^2^, ^3^ Moreover, dapagliflozin and empagliflozin alleviated HF symptoms and improved physical function and quality of life.^48^, ^49^ Benefits were seen early after the initiation of the SGLT2i. The effects were similar, with no significant interaction, in patients with and without diabetes and across the whole spectrum of glycated haemoglobin (HbA1c) values.

**Table 2:** Major outcome trials of SGLT2is in patients with heart failure.

Study name and year	Drug comparisons	Number of pts	Main eligibility criteria	Primary outcome	Effect of treatment on primary outcome HR; 95% CI	Effect of treatment on primary outcome Rate per 100 patient‐years (SGLT2i vs. placebo)
DAPA‐HF, 2019^2^	Dapagliflozin (10 mg once daily) or placebo	4744	≥18 years old HF with LVEF ≤ 40% NYHA II–IV Elevated NT‐proBNP On HFrEF therapy SBP ≥ 95 mmHg eGFR ≥ 30 mL/min/1.73 m^2^	CV death or worsening HF (hospitalization or an urgent visit resulting in intravenous therapy for HF)	0.74; 0.65–0.85	11.6 vs. 15.6
EMPEROR‐Reduced, 2020^3^	Empagliflozin (10 mg once daily) or placebo	3730	≥18 years old LVEF ≤ 40% NYHA II–IV Elevated NT‐proBNP If LVEF > 30%, a history of HFH within 12 months or higher NT‐proBNP required On HFrEF therapy SBP ≥ 100 mmHg eGFR ≥ 20 mL/min/1.73 m^2^	CV death or hospitalization for worsening HF	0.75; 0.65–0.86	15.8 vs. 21.0
EMPEROR‐Preserved, 2021^7^, ^43^, ^44^, ^45^	Empagliflozin (10 mg once daily) or placebo	5988	≥18 years old HF with LVEF > 40% NYHA II–IV Elevated NT‐proBNP Structural heart disease within 6 months or documented HFH within 12 months eGFR ≥ 20 mL/min/1.73 m^2^	CV death or HFH	0.79; 0.69–0.90	6.9 vs. 8.7
DELIVER, 2022^8^, ^46^, ^47^	Dapagliflozin (10 mg once daily) or placebo	6263	≥40 years old Stabilized HF with LVEF > 40% Outpatients or during HFH NYHA II–IV Structural heart disease (either left atrial enlargement or LV hypertrophy) Elevated NT‐proBNP HFimpEF was eligible eGFR ≥ 25 mL/min/1.73 m^2^	Composite of worsening HF (either an unplanned HFH or an urgent visit for HF) or CV death	0.82; 0.73–0.92	7.8 vs. 9.6

Abbreviations: CI, confidence interval; CV, cardiovascular; eGFR, estimated glomerular filtration rate; HF, heart failure; HFH, hospitalization for heart failure; HFimpEF, heart failure with improved ejection fraction; HFrEF, heart failure with reduced ejection fraction; HR, hazard ratio; LV, left ventricular; LVEF, left ventricular ejection fraction; NT‐proBNP, N‐terminal pro‐brain natriuretic peptide; NYHA, New York Heart Association; pts, patients; SBP, systolic blood pressure; SGLT2i, sodium–glucose cotransporter 2 inhibitor.

Beneficial effects were mainly driven by the reduction in HF hospitalizations. The relative risk reduction in all‐cause mortality was smaller compared with pivotal trials with neurohormonal modulators. However, reduction in mortality, compared with previous decades, may reduce the likelihood of influencing this endpoint with new drugs.

The ARR of major events may be interpreted differently, but it was, in any case, larger than in the trials in patients with diabetes or CKD.^37^, ^50^ Reduction in all‐cause deaths and CV deaths reached statistical significance in DAPA‐HF but not in EMPEROR‐Reduced, though with no heterogeneity between the two trials.^51^ The ARR ranged from 1.6 to 0.5 all‐cause and CV deaths per 100 patient‐years, lower than the values found in most of the neurohormonal modulators trials.^50^, ^52^, ^53^ Larger values were found for HF hospitalizations alone, which were reduced by 30%–31% (HR 0.70; 95% CI 0.59–0.83 in DAPA‐HF and HR 0.69; 95% CI 0.59–0.81 in EMPEROR‐Reduced) with an ARR of 3.7 and 4.8, respectively.^2^, ^3^, ^51^

Based on the results of DAPA‐HF and EMPEROR‐Reduced, dapagliflozin or empagliflozin was recommended in patients with HFrEF to reduce HF hospitalizations and CV death, regardless of diabetes status.^2^, ^3^, ^5^, ^6^ Including the results of SOLOIST‐WHF, which enrolled patients with diabetes and worsening HF, sotagliflozin was also recommended to improve outcomes in patients with HFrEF and T2DM.^5^, ^54^

No further major outcome trial with SGLT2is in outpatients with HFrEF was conducted after publication of the ESC HF guidelines. However, several secondary analyses of DAPA‐HF and EMPEROR‐Reduced trials, as well as pooled analyses of these trials, were published.^55^, ^56^, ^57^, ^58^, ^59^, ^60^, ^61^, ^62^ These secondary analyses generally corroborated the consistency of treatment benefits across several subgroups of patients, stratified by demographic characteristics (e.g., sex and age),^55^, ^59^, ^63^ comorbidities and concomitant treatments.^56^, ^60^ A meta‐analysis suggested treatment‐by‐subgroup interactions for subgroups based on New York Heart Association (NYHA) functional class, race and regions.^51^ The pooled HR for patients in NYHA Class II differed from that for patients in Class III–IV with an attenuated effect reported in patients with more severe HF symptoms (interaction *P* = 0.0087).^51^ With respect of race, the effects on the primary outcome were smaller in the white patients (HR 0.83; 95% CI 0.74–0.93), compared with black and Asian ones (HR 0.53; 95% CI 0.37–0.76 and HR 0.61; 95% CI 0.49–0.75, respectively; *P* = 0.0063 for subgroup interactions). Similarly, SGLT2i effects were larger in North and Latin American and Asian patients than in European ones.^50^, ^51^ These results are hypothesis generating and would deserve appropriate trials, which are, however, unlikely to occur.

### HF with mildly reduced ejection fraction (HFmrEF) and HFpEF

Post hoc analyses of CANVAS and DECLARE‐TIMI 58 suggested that patients with diabetes and HFpEF might have benefited from treatment with SGLT2is. However, in these analyses, only a small number of events were reported.^64^, ^65^ Pre‐specified subgroup analyses of the SOLOIST‐WHF trial showed consistent treatment effect across the LVEF spectrum, but the early termination of the trial and the small sample size limited the interpretation of these results.^54^

EMPEROR‐Preserved and, 1 year later, DELIVER showed the efficacy of SGLT2 inhibition also in patients with HFmrEF and HFpEF.^43^, ^46^, ^66^ Detailed inclusion criteria for each trial are reported in Table 2. Empagliflozin reduced the combined risk of CV death or hospitalization for HF (HR 0.79; 95% CI 0.69–0.90; ARR 1.8 events per 100 patient‐years), irrespective of diabetes history.^7^ Similarly, dapagliflozin significantly reduced the primary composite endpoint of CV death or worsening HF event (either an HF hospitalization or an urgent HF visit) (HR 0.82; 95% CI 0.73–0.92; ARR 1.8 events per 100 patient‐years). There was a significant reduction only in HF hospitalizations and not in CV death alone in each single trial. Overall, SGLT2is prevented 5–8 HF hospitalizations per 100 patient‐years in HFrEF trials and 2–3 worsening HF events per 100 patient‐years of treatment in HFpEF trials.^53^ The results were consistent regardless of the baseline glycaemic status,^67^ duration of HF,^68^ baseline LVEF,^69^ history of reduced ejection fraction with subsequent improvement (HFimpEF),^70^ background medical therapy^61^, ^71^, ^72^, ^73^ and multiple other clinically relevant variables.^69^

Using study‐level published data from EMPEROR‐Reduced, EMPEROR‐Preserved and SOLOIST‐WHF and participant‐level data from DAPA‐HF and DELIVER with harmonized endpoint definitions, a pre‐specified and pre‐registered meta‐analysis of these trials (*n* = 21 947) confirmed the efficacy of SGLT2is in patients with HF irrespective of LVEF.^69^ Overall, SGLT2is reduced the risk of CV death or hospitalization for HF (HR 0.77; 95% CI 0.72–0.82), with an NNT of 25 (20–31) over a weighted mean of 23 months' follow‐up. The risk of a first hospitalization for HF was reduced [HR 0.72; 95% CI 0.67–0.78; NNT 28 (24–35)]. The meta‐analysis also showed a reduction in CV death [HR 0.87; 95% CI 0.79–0.95; NNT 88 (54–229)] and death from any cause [HR 0.92; 95% CI 0.86–0.99; NNT 92 (52–733)].^69^ Of note, there was no evidence of between‐trial heterogeneity of treatment effect for any of these outcomes.^69^ The effect of SGLT2is on the composite of CV death or first HF hospitalization was consistent across 14 clinically relevant subgroups with no heterogeneity except for NYHA functional classification, with an attenuation of effect in patients with NYHA Class III or IV (HR 0.86; 95% CI 0.77–0.95) compared with those with NYHA Class II (HR 0.72; 95% CI 0.67–0.79); *P* value for heterogeneity 0.015.^69^

### Background diuretic therapy

Changes in diuretic therapy may be an index of HF severity, with the need to increase diuretic doses that may be regarded as a worsening HF event, consistent with poorer outcomes.^74^, ^75^ SGLT2is were associated with a meaningful reduction of both inpatient and outpatient worsening HF events.^44^, ^47^, ^74^ Dapagliflozin significantly reduced new initiation of loop diuretics in DELIVER.^47^ First sustained loop diuretic dose increases were less frequent, and sustained dose decreases were more frequent in patients treated with empagliflozin in EMPEROR‐Preserved.^44^ The mean dose of loop diuretic increased over time in the placebo arm, whereas this was attenuated with SGLT2i treatment. The reduction in diuretic need after the initiation of SGLT2is might be the consequence of both renal and cardiac long‐term benefits.^50^, ^61^, ^76^

### Quality of life and exercise capacity

SGLT2is improved symptoms and quality of life, mainly measured using the Kansas City Cardiomyopathy Questionnaire (KCCQ), in major RCTs.^69^, ^77^, ^78^, ^79^, ^80^, ^81^, ^82^ A meta‐analysis of 14 RCTs, including 21 737 patients, showed a significant improvement in the Kansas City Cardiomyopathy Questionnaire Overall Summary Score (KCCQ‐OSS) of 1.94 points (95% CI 1.41–2.46) at 3 months and of 2.18 points (95% CI 1.13–3.24) at 6 months from baseline with SGLT2i compared with placebo, irrespective of LVEF.^82^ It has been argued about the clinical significance of these results, as the difference from placebo was rather small, with statistical significance influenced by the large size of the study groups. Second, a consistent proportion of patients had a good quality of life at baseline with, therefore, virtually no room for further improvement.^53^ It must be, however, also considered that the value of such relatively small changes is greater as collected in the context of a double‐blind trial compared with data from unblinded trials or with medications affecting other objective and easily detectable parameters, such as body weight.^53^, ^83^, ^84^, ^85^

Studies focused on the assessment of the effects of SGLT2is on quality of life and exercise capacity have been more recently published. DETERMINE (Dapagliflozin Effect on Exercise Capacity Using a 6‐Minute Walk Test in Patients With Heart Failure) was a double‐blind, placebo‐controlled, multicentre trial assessing the efficacy of dapagliflozin on symptoms, quality of life and exercise capacity in patients with HFrEF and HFpEF. Dapagliflozin administration was associated with a significant improvement in the Kansas City Cardiomyopathy Questionnaire Total Symptoms Score (KCCQ‐TSS) at 16 weeks in patients with HFrEF (4.2; 95% CI 1.0–8.2; *P* = 0.022) but not in patients with HFpEF. Also, the 6 min walking test distance (6MWTD) tended to improve with dapagliflozin but without reaching statistical significance versus placebo.^86^ In the double‐blind PRESERVED‐HF trial (Effects of Dapagliflozin on Biomarkers, Symptoms and Functional Status in Patients With Preserved Ejection Fraction Heart Failure), patients randomized to dapagliflozin were more likely to show an improvement in the 6MWTD and less likely to have its deterioration, compared with placebo.^87^

### Invasive haemodynamics

CAMEO‐DAPA (Cardiac and Metabolic Effects of Dapagliflozin in HFpEF) was a single‐centre, double‐blind, randomized, placebo‐controlled trial testing the effects of dapagliflozin on invasive haemodynamic parameters at rest and during exercise at baseline and after 24 weeks.^88^ Dapagliflozin administration reduced pulmonary capillary wedge pressure (primary endpoint) at rest [estimated treatment difference −3.5 mmHg (95% CI −6.6 to −0.4); *P* = 0.029] and maximal exercise [−5.7 mmHg (95% CI −10.8 to −0.7); *P* = 0.027].^88^ Further analyses showed an improvement in arterial compliance, an increase in venous capacitance and a better ventilatory control during exercise with dapagliflozin administration.^89^, ^90^

Other studies were based on patients with a previously implanted pulmonary artery pressure sensor (CardioMEMS) and showed a significant improvement in haemodynamic parameters, namely, a reduction in pulmonary artery diastolic pressure with SGLT2is versus placebo. Results were consistent regardless of LVEF and at different time points (from 8–12 to 24 weeks).^91^, ^92^ The size effect seems, however, small [−1.5 (95% CI −0.2–2.8) mmHg lower at Weeks 8–12].^92^

### SGLT2i withdrawal

Packer *et al*. reported the results of a pre‐specified analysis of the effects of blinded withdrawal of empagliflozin in the EMPEROR‐Reduced and EMPEROR‐Preserved trials.^93^ Compared with the 90 days prior to the end of the study, during the first 30 days after drug withdrawal, the annualized risk of CV death or hospitalization for HF increased in patients withdrawn from empagliflozin but not in those withdrawn from placebo (from 10.74 to 17.04 events per 100 patient‐years versus from 13.47 to 14.11 events per 100 patient‐years in patients who were receiving empagliflozin and placebo, respectively; HR 1.75; 95% CI 1.20–2.54). Consistently, the Kansas City Cardiomyopathy Questionnaire Clinical Summary Score (KCCQ‐CSS) declined by 1.6 ± 0.4 in patients withdrawn from empagliflozin versus placebo (*P* < 0.0001). Withdrawal of empagliflozin was accompanied by increases in fasting glucose, body weight, systolic blood pressure, eGFR, NT‐proBNP, uric acid and serum bicarbonate and decreases in haemoglobin and haematocrit (all *P* < 0.01).^93^ These dramatic changes mirrored the early effects of treatment ≈1–3 years earlier in the same group of patients. Though they cannot clarify the mechanism of action of SGLT2is, they show that it is short‐lived with the need to maintain treatment in the long term.

## Comorbidities

Patients with HF suffer from a range of comorbidities, which have an impact on outcomes and quality of life and affect the prescription of medical therapy.^5^, ^9^, ^94^, ^95^

### Obesity and adiposity

Obesity is very common in contemporary HFpEF cohorts. Up to a 96% prevalence of central obesity, assessed by the waist‐to‐height ratio, was reported in PARAGON‐HF.^96^ There was a 45% prevalence of obesity in the DELIVER and EMPEROR‐Preserved trials. The treatment effect of dapagliflozin and empagliflozin versus placebo was consistent across all the body mass index (BMI) and waist‐to‐height ratio categories with no significant interaction.^97^, ^98^ Health‐related quality of life improved, and body weight fell in all BMI categories, with greater improvement in more obese patients.^97^, ^98^ The weight loss with SGLT2is is generally moderate (on average 1–2 kg placebo‐adjusted weight loss) due to counter‐regulatory mechanisms striving to maintain body weight.^99^ In patients with obesity, SGLT2is might be combined with other agents having anorexigenic effects, for example, GLP‐1 receptor agonists (GLP‐1RAs) that have demonstrated favourable effects on body weight, quality of life, exercise capacity and possibly outcomes in patients with HFpEF and obesity.^100^, ^101^, ^102^ Trials in patients with T2DM show that the beneficial effects of GLP‐1RAs on CV events and HF hospitalizations are maintained regardless of SGLT2i use with a possibility of synergistic effects through complementary mechanisms of action.^15^, ^103^

### Hypertension and hypotension

A potential antihypertensive effect of SGLT2is has been proposed. However, it seems to be present only in patients with higher blood pressure at baseline, whereas changes in blood pressure are minimal in patients with lower blood pressure.^104^, ^105^ Participants from DELIVER and EMPEROR‐Preserved with treatment‐resistant hypertension had the highest rate of the primary outcome with a similar, if not larger, risk reduction with dapagliflozin or empagliflozin compared with patients with non‐resistant hypertension and controlled blood pressure.^106^, ^107^

Hypotension is often a major limitation to the implementation of medications acting through neurohormonal mechanisms. A systolic blood pressure ≤95–100 mmHg was generally an exclusion criterion in the major HF trials with dapagliflozin and empagliflozin, and we, thus, have no data for these patients. Changes in blood pressure were, however, minimal, in the range of a small 1–2 mmHg, compared with placebo, in patients with the lowest blood pressure at baseline in HF trials, making the administration of SGLT2is well tolerated and supporting their early and safe initiation also in patients not tolerating renin–angiotensin system (RAS) inhibitors for hypotension.^108^, ^109^, ^110^, ^111^, ^112^, ^113^, ^114^

Treatment effects of both dapagliflozin and empagliflozin were consistent irrespective of baseline systolic blood pressure across all trials in patients with HF.^106^, ^107^, ^108^, ^109^, ^110^, ^111^

### CKD

All HF trials showed a slower decline in eGFR with the SGLT2is compared with placebo.^2^, ^3^, ^7^, ^8^ Beneficial effects on CV and renal outcomes, as well as on the slope of eGFR decline, were shown across subgroups of patients with HF and with or without comorbid CKD.^115^, ^116^, ^117^, ^118^, ^119^ Improvement in kidney function may allow better tolerance of other evidence‐based therapies for HF, such as MRA.^120^, ^121^

An early eGFR reduction due to reduced intraglomerular pressure has been consistently shown after SGLT2i initiation. However, it had no untoward relation with outcome.^122^, ^123^

An eGFR ≤ 20–30 mL/min/1.73 m^2^ of body surface area was an exclusion criterion in major HF trials. Thus, dapagliflozin or empagliflozin cannot be recommended in patients with the lowest eGFR.^5^, ^9^ However, retrospective analyses support prosecution of SGLT2i administration even in patients showing deterioration of kidney function. In a participant‐level pooled analysis of the DAPA‐HF and DELIVER trials, Chatur *et al*. evaluated the associations between a decline of eGFR to <25 mL/min/1.73 m^2^, dapagliflozin administration, efficacy and safety outcomes.^124^ Patients with a deterioration of kidney function to an eGFR of <25 mL/min/1.73 m^2^ had a heightened risk of subsequent events. Prosecution of dapagliflozin administration was associated with lower rates of the primary composite outcome with a similar safety also in these patients experiencing a deterioration of kidney function.^124^

With respect to other comorbidities, treatment benefits of SGLT2is were not affected by baseline history of diabetes and background glucose‐lowering therapy, peripheral artery disease, chronic obstructive pulmonary disease or liver disease.^60^, ^62^, ^69^, ^125^, ^126^

## Acute HF

Evidence from recent trials supports the early initiation of SGLT2is in patients with recent HF decompensation or during a hospitalization due to acute HF.^74^, ^113^, ^127^, ^128^ This indication is supported by the analysis of patients starting treatment while hospitalized or early after discharge in DELIVER,^129^ by the post hoc analysis of SOLOIST‐WHF of patients starting sotagliflozin before discharge for worsening HF^130^ and by the EMPULSE (Empagliflozin in Patients Hospitalized with Acute Heart Failure Who Have Been Stabilized) trial. The EMPULSE trial demonstrated a benefit on the combined endpoint of quality of life and outcomes with empagliflozin administration, versus placebo, in patients recently hospitalized for HF.^131^ Clinical benefit was seen as early as at 15 days and maintained through 90 days and was achieved regardless of the degree of symptomatic impairment at baseline.^132^ Further trials in acute HF are summarized in Table S1.^133^, ^134^, ^135^ In a secondary analysis of the EMPULSE trial, Biegus *et al*. showed that empagliflozin, compared with placebo, significantly reduced all the studied markers of decongestion, including weight loss, weight loss adjusted for mean daily loop diuretic dose, area under the curve of change from baseline in NT‐proBNP levels, haemoconcentration and clinical congestion score at all time points.^136^

Potential decongestive mechanisms of SGLT2is might include osmotic diuresis, natriuresis, preservation of glomerular filtration and facilitation of interstitial drainage, which can collectively translate into effective and safe decongestion.^137^, ^138^ Parallel vasopressin release and renal water reabsorption may, however, quickly counterbalance the osmotic diuresis leading to increased urine concentration with thereby urine volume stabilization.^139^ Thus, the limited diuretic response to SGLT2is results from a rapid renal adaptation to these drugs that is nearly complete within a few days or weeks, preventing a persistent effect.^140^ Additional mechanisms, including urinary loss of calories as a result of glycosuria and stimulation of erythropoietin production and erythrocytosis, have also been advocated to explain loss of weight and the increase in haematocrit.^56^, ^134^, ^141^

The recently published DICTATE‐AHF trial randomized 240 patients within 24 h of hospital presentation for hypervolaemic acute HF to dapagliflozin 10 mg once daily or structured usual care diuretic titration. The primary outcome of diuretic efficiency, defined as cumulative weight change per cumulative loop diuretic dose, was not significantly different between dapagliflozin and usual care [odds ratio (OR) 0.65; 95% CI 0.41–1.02; *P* = 0.06]. Dapagliflozin was associated with a reduction in loop diuretic doses and fewer intravenous diuretic up‐titrations to achieve decongestion.^142^

Dapagliflozin was also tested in patients admitted due to acute HF and with diuretic resistance, defined as an inadequate response to an intravenous loop diuretic.^74^, ^143^ DAPA‐RESIST was a multicentre, open‐label, randomized trial comparing dapagliflozin 10 mg or metolazone 5–10 mg once daily for 3 days in patients hospitalized for acute HF and with diuretic resistance. Diuretic effect, assessed as change in body weight at Day 5, was not different, but patients assigned to dapagliflozin received a larger cumulative dose of furosemide, resulting in a lower diuretic efficiency.^137^, ^144^

A real‐world analysis using linkable administrative databases in Ontario, Canada, supports early SGLT2i initiation to improve prognosis in acute HF.^145^ Other smaller studies were published,^146^ and further ongoing studies may provide additional evidence as regards the optimal timing for early initiation of SGLT2is in patients hospitalized due to acute HF with volume overload.^147^

## Valvular heart disease and other structural heart diseases

### Aortic stenosis

Patients with valvular heart disease, including those undergoing transcatheter aortic valve implantation (TAVI), have been excluded from SGLT2i randomized trials. Raposeiras‐Roubin *et al*. conducted an RCT to evaluate the efficacy of dapagliflozin, as compared with standard care alone, in 1222 patients with aortic stenosis who were undergoing TAVI and had a history of HF plus at least one of the following: renal insufficiency, diabetes or LV systolic dysfunction.^148^, ^149^ At 1 year follow‐up, death from any cause or worsening HF, defined as hospitalization or an urgent visit, the primary outcome, occurred in 91 patients (15.0%) in the dapagliflozin group versus 124 patients (20.1%) in the standard‐care group (HR 0.72; 95% CI 0.55–0.95). Reduction in all‐cause death did not reach statistical significance (HR 0.87; 95% CI 0.59–1.28), whereas worsening HF events were less with dapagliflozin (HR 0.63; 95% CI 0.45–0.88; ARR 5%).^149^

### Cardiomyopathies and congenital heart disease

Patients with infiltrative cardiomyopathies and with congenital heart disease were excluded from RCTs. Thus, evidence regarding their use in this specific setting is still scarce and based on observational studies.

In a retrospective analysis of 2356 consecutive patients with transthyretin amyloid cardiomyopathy (ATTR‐CM), administration of SGLT2is was associated with less worsening of NYHA functional class, NT‐proBNP and eGFR values; fewer new initiations of loop diuretics; and lower risk of HF‐related events at 12 months at propensity score analysis.^150^ Results in ATTR‐CM were confirmed in an independent study.^151^ Discontinuation rate for SGLT2i in this population ranged between 4.5% and 11.5% and was most commonly due to genitourinary symptoms.^150^, ^151^

The SONATA‐HCM (NCT06481891) is a phase 3, randomized, double‐blind, placebo‐controlled multicentre trial that is evaluating the efficacy of sotagliflozin on symptoms, functional capacity and other patient‐reported outcomes, as well as safety in patients with symptomatic hypertrophic cardiomyopathy (HCM).

Initial experience with SGLT2is in paediatric patients shows their good tolerability and possible efficacy. Larger studies are needed to evaluate them in this population.^152^ Neijenhuis *et al*. reported the safety, tolerability and short‐term HF‐related benefits of SGLT2is in a real‐world population of adults with congenital heart disease (ACHD).^153^

**Figure 2:**
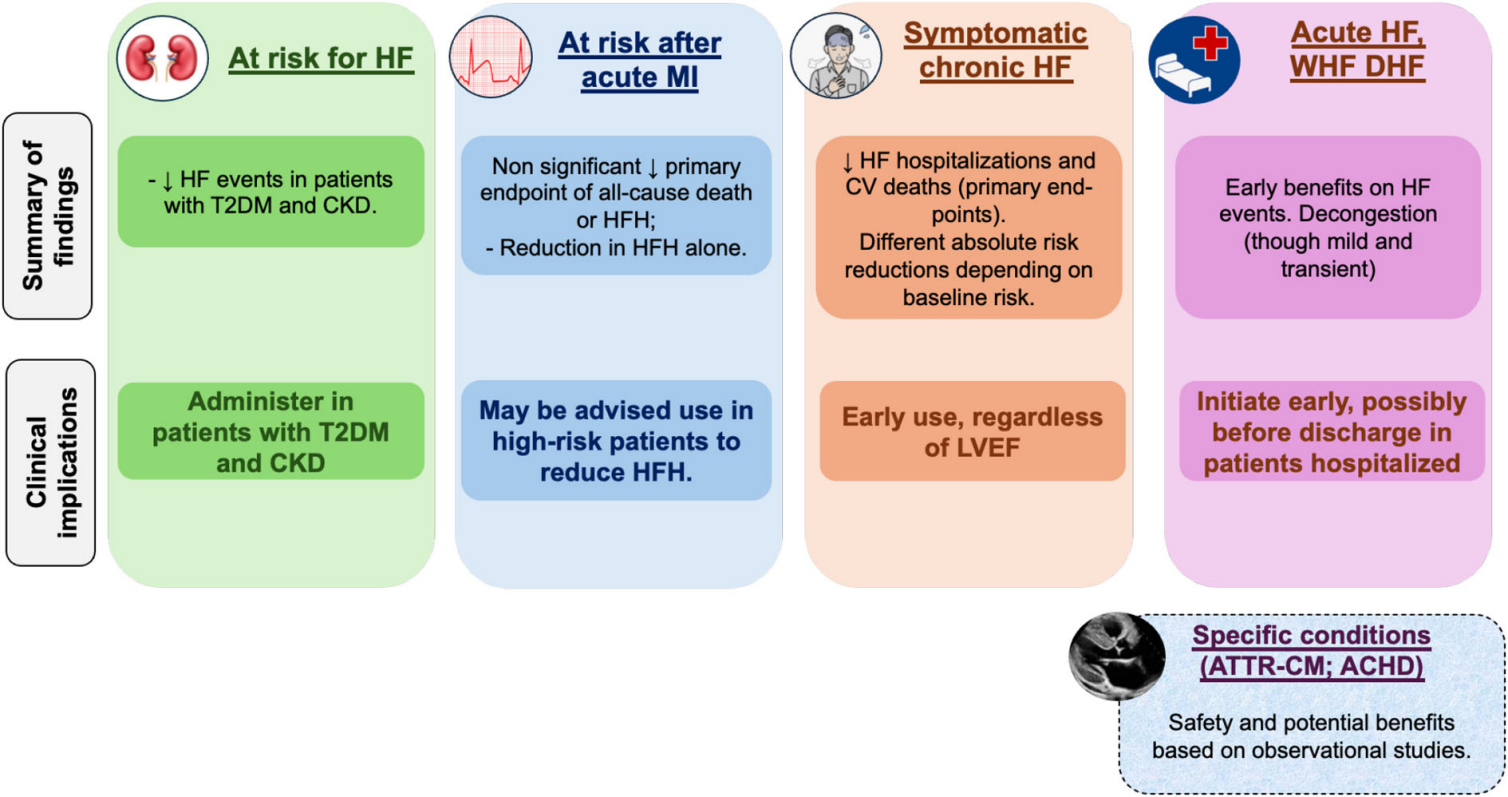
Evidence supporting the use of sodium–glucose cotransporter 2 inhibitors across different heart failure (HF) stages and phenotypes.ACHD, adults with congenital heart disease; ATTR‐CM, transthyretin amyloid cardiomyopathy; CKD, chronic kidney disease; CV, cardiovascular; DHF, decompensated HF; HFH, HF hospitalizations; LVEF, left ventricular ejection fraction; MI, myocardial infarction; RCT, randomized controlled trial; T2DM, type 2 diabetes mellitus; TAVR, transcatheter aortic valve replacement; WHF, worsening HF.

These data provide a rationale to extend the use of SGLT2i in patients with cardiac amyloidosis and ACHD (Figure 2), but prospective randomized data are needed to confirm safety and to prove efficacy in these specific groups of patients.

## Mechanistic trials

Mechanisms underlying the positive effects of SGLT2is have been extensively reviewed.^20^, ^137^, ^154^, ^155^, ^156^, ^157^, ^158^ The effects of SGLT2is on cardiac remodelling and myocardial function were studied in relatively few trials.^159^, ^160^ A total of 162 patients with stable HF (52% with LVEF > 40%) were enrolled in the DAPA‐MODA trial. Left atrial volume index (LAVI) showed a significant reduction at 180 days, primarily due to a decrease in reservoir volume; LV geometry improved with significant reductions in LV mass index, end‐diastolic volume and end‐systolic volume.^159^ An early remodelling of the left atrium due to acute dynamic changes in left heart filling parameters has been described after 5 days of treatment with empagliflozin versus placebo among patients hospitalized with acute decompensated HF.^161^ A long‐term favourable effect of SGLT2is on LV remodelling has been previously described in patients with T2DM and/or HFrEF,^160^, ^162^, ^163^ but not in the EMPA‐HEART 2 CardioLink‐7 randomized clinical trial, including individuals with risk factors for adverse cardiac remodelling but neither diabetes nor significant HF.^164^ A meta‐analysis of six trials, including 555 patients, suggested LV reverse remodelling with an improvement in LV volumes, LVEF and LAVI in patients with HFrEF receiving SGLT2is. Reduction in LV mass index did not reach statistical significance, and no significant change in LV global longitudinal strain was noted.^165^

Several clinical studies have shown that SGLT2is promote erythrocytosis and reduce inflammation.^56^, ^141^, ^166^, ^167^ Transferrin saturation, ferritin and hepcidin were reduced, and total iron‐binding capacity and soluble transferrin receptor increased with SGLT2is compared with placebo.^167^

EMPA‐VISION (Assessment of Cardiac Energy Metabolism, Function and Physiology in Patients With Heart Failure Taking Empagliflozin) failed to demonstrate a significant change in myocardial energetics as assessed with cardiac phosphocreatine:ATP ratio (PCr/ATP) at rest and during peak dobutamine stress. Likewise, no changes in serum metabolomics or levels of circulating ketone bodies were observed.^168^

SGLT2is likely act directly, even independently from the presence of SGLT2 receptors, on the myocardial cells with a down‐regulation of nutrient surplus and an up‐regulation of nutrient deprivation signalling pathways and of autophagy with an increase in the expression and activity of adenosine monophosphate‐activated protein kinase (AMPK), sirtuin 1 (SIRT1), sirtuin 3 (SIRT3), sirtuin 6 (SIRT6) and peroxisome proliferator‐activated receptor γ coactivator 1‐α (PGC1‐α) and decreased activation of mammalian target of rapamycin (mTOR). Such mechanisms are likely able to improve myocardial function even in the short term with, however, rapid loss of efficacy after their withdrawal.^93^, ^155^, ^169^

## Implications for clinical practice

Based on the results of major RCTs and the lack of heterogeneity in meta‐analyses, dapagliflozin and empagliflozin are now advised for the treatment of all the patients with HF independently from their LVEF and diabetes status.^5^, ^9^ No significant difference that could justify the use of one drug over the other was found. To date, sotagliflozin is approved in the European Union for the treatment of type 1 diabetes and in the United States, since May 2023, to reduce the risk of death due to HF and, possibly, CV events in patients with T2DM.

The time needed to reach statistical significance of a clinical effect is very dependent on the size of the study group. However, Kaplan–Meier curves for time‐to‐first‐event analysis diverge early in SGLT2i trials,^170^ and they are effective and well tolerated also when initiated in‐hospital in patients with acute decompensation of HF after stabilization.^131^, ^136^, ^171^ It is therefore now advised to start them early once the patients receive a diagnosis of HF or experience an episode of decompensation.^9^, ^113^, ^127^

Due to the safety profile, good tolerability, ease of administration and efficacy alongside the entire spectrum of HF, SGLT2is represent first‐line therapy for HF patients, regardless of LVEF (Figure 2).^9^, ^58^, ^69^, ^172^ Although the relative risk reduction in all‐cause mortality is much smaller in current SGLT2i trials compared with that achieved in the past trials with beta‐blockers and MRAs,^52^, ^53^ these agents still cause a significant reduction in HF hospitalizations and improve quality of life,^82^ all very important aspects for the patient's clinical journey.^173^

A low eGFR (<20–25 mL/min/m^2^) and, possibly, a low systolic blood pressure (<95–100 mmHg) seem to be the main limitations to their use based on the inclusion criteria of major RCTs. Nevertheless, secondary analyses of these trials showed a minimal decrease in systolic blood pressure after SGLT2i initiation, potentially extending the treatment to hypotensive patients. Urinary tract infection is the most frequent adverse event and cause of discontinuation. Importantly, caution is needed with SGLT2is in patients with type 1 diabetes.^27^ Adding an SGLT2i at a lower than usual dose to insulin therapy in these patients may facilitate glucose control and decrease glucose excursions, thereby reducing insulin doses and hypoglycaemia.^174^ However, SGLT2i therapy increased the risk of diabetic ketoacidosis.^174^ Ketoacidosis at lower glucose levels, so‐called ‘euglycaemic ketoacidosis’, has been reported in 2%–3% of patients with type 1 diabetes taking SGLT2is.^175^

## Implementation in clinical practice

There is now a major indication for SGLT2is in patients with HF, irrespective of their LVEF.^9^

A recent analysis of the Swedish HF registry showed that eligibility to treatment with dapagliflozin or empagliflozin applies to up to 80% of patients with HFrEF and 75% of patients with HFmrEF and HFpEF, respectively.^176^ Using data from the Global Burden of Disease 2017 report, Talha *et al*. estimated that ~50 million patients with HF have an indication to SGLT2is, with almost half of them with an LVEF > 40%. Based on the results of clinical trials, administration of these medications would prevent 7–8 million worsening HF events and CV deaths over 3 years with a major saving on healthcare costs.^177^ Similar data were obtained in an analysis of the National Health and Nutrition Examination Survey.^178^

The rate of use of SGLT2is in clinical practice remains, however, suboptimal. Treatment of the patients recently hospitalized for HF was analysed in EVOLUTION HF (Utilization of Dapagliflozin and Other Guideline Directed Medical Therapies in Heart Failure Patients: A Multinational Observational Study Based on Secondary Data). Mean times from the day of hospitalization to initiation of guideline‐directed medical therapies (GDMTs) were longer for novel medications (SGLT2is or sacubitril/valsartan) than for other GDMTs. Twenty‐three per cent of the patients discontinued SGLT2is during 12 months of follow‐up with better persistence compared with other GDMTs.^179^

Stolfo *et al*. showed a three‐fold increase, from 20.5% to 59.0%, in the administration of SGLT2is in patients with HFrEF enrolled in the Swedish HF registry between 1 November 2020 and 5 August 2022. Interestingly, the steeper increase in SGLT2i use occurred after the release of the 2021 ESC HF guidelines, which introduced them among the four pillars for the treatment of HFrEF. Discontinuation rate at 6 and 12 months was 13.1% and 20.0%, respectively.^180^ Thus, the adoption in clinical practice seems more rapid, and the discontinuation rate was lower with SGLT2is than with the other traditional medications for HF. However, these results still remain largely suboptimal, although due to the single‐dose application and good tolerability, their implementation appears to be unproblematic. Poor socioeconomic support, limitations in the organization of HF management, non‐specialist care and clinical inertia still play a major role.^177^, ^179^, ^180^, ^181^, ^182^

## Conclusions

We have summarized recent data that prompted a change in current indications to SGLT2is for the prevention and treatment of HF across various patient populations.^22^ The reduction in HF events with SGLT2is in patients with CKD and T2DM further expanded their indication for the prevention of HF in these patients. More consistently, based on the results of primary endpoints of major clinical trials, empagliflozin and dapagliflozin improved outcomes in patients with HF irrespective of LVEF and are recommended for the treatment of HF across the entire LVEF spectrum in the 2023 ESC HF guidelines. Additive and synergistic effects of SGLT2is with both MRAs and GLP‐1RA support the combination therapy across the entire cardiovascular–kidney–metabolic spectrum.

## Conflict of interest statement

M.M. reports personal consulting honoraria of minimal amount from Abbott, Amgen, Bayer, Edwards Lifesciences, LivaNova and Vifor Pharma for participation in advisory board meetings and executive committees of clinical trials outside the submitted work. D.T. reports speaker fees and honoraria of minimal amount from Alnylam Pharmaceuticals, AstraZeneca, Boehringer Ingelheim, Pfizer and Roche Diagnostics outside the submitted work. M.A. reports speaker fees from Abbott and Medtronic outside the submitted work. O.A. reports consulting fees from Sensible Medical Innovations Ltd. S.D.A. reports grants and personal fees from Vifor and Abbott and personal fees for consultancies, trial committee work and/or lectures from Actimed, Amgen, AstraZeneca, Bayer, Boehringer Ingelheim, BioVentrix, Brahms, Cardiac Dimensions, Cardior, Cordio, CVRx, Cytokinetics, Edwards Lifesciences, Faraday Pharmaceuticals, GSK, HeartKinetics, Impulse Dynamics, Novartis, Occlutech, Pfizer, Repairon, Sensible Medical, Servier, Vectorious and V‐Wave and was named co‐inventor of two patent applications regarding MR‐proANP (DE 102007010834 and DE 102007022367), but he does not benefit personally from the related issued patents. A.B‐.G. has received speaker honoraria and/or consulting for AstraZeneca, Bayer, Boehringer Ingelheim, Novartis, Roche Diagnostics and Vifor. M.B. is supported by the Deutsche Forschungsgemeinschaft (German Research Foundation; TTR 219, Project Number 322900939) and reports personal fees from Abbott, Amgen, AstraZeneca, Bayer, Boehringer Ingelheim, Cytokinetics, Medtronic, Novartis, Recor Medical, Servier and Vifor. J.B. consulting honoraria from Abbott, American Regent, Amgen, Applied Therapeutic, AskBio, Astellas, AstraZeneca, Bayer, Boehringer Ingelheim, Boston Scientific, Bristol Myers Squibb, Cardiac Dimensions, Cardiocell, Cardior, CSL Bearing, CVRx, Cytokinetics, Daxor, Edwards Lifesciences, Element Science, Faraday Pharmaceuticals, Foundry, G3P, Innolife, Impulse Dynamics, Imbria, Inventiva, Ionis, Lexicon, Lilly, LivaNova, Janssen, Medtronic, Merck, Occlutech, Owkin, Novartis, Novo Nordisk, Pharmacosmos, Pharmain, Pfizer, Prolaio, Regeneron, Renibus, Roche, Salamandra, Sanofi, SC Pharma, Secretome, Sequana, SQ Innovation, Tenex, Tricog, Ultromics, Vifor and Zoll outside the submitted work. O.C. reports Servier travel support for ESC Congress. G.F. reports lecture fees and/or committee member contributions in clinical trials sponsored by Bayer, Medtronic, Vifor, Servier, Novartis, Amgen and Boehringer Ingelheim and research support from the European Union. F.G. reports consulting fees for Bayer, Alnylam Pharmaceuticals, Ionis, Pfizer and Abbott and speaker fees from AstraZeneca, Novartis and Orion Pharma. E.A.J. reports research grants and personal fees from Vifor Pharma and personal fees from Bayer, Novartis, Abbott, Boehringer Ingelheim, Pfizer, Servier, AstraZeneca, Berlin Chemie, Cardiac Dimensions, Takeda and Gedeon Richter. J.C.K. reports speaker honoraria from Menarini and Servier UK. B.M. reports advisory or speakers fees from AstraZeneca, Bayer, Boehringer Ingelheim, Eli Lilly, Novartis, Servier, Vifor Pharma, Menarini and Merck Serono. M.C.P. reports research grants from SQ Innovation, AstraZeneca, Roche, Boehringer Ingelheim, Pharmacosmos, Eli Lilly, Napp Pharmaceuticals, Novartis and Novo Nordisk; has served on committees for AbbVie, Akero, Alnylam Pharmaceuticals, AstraZeneca, Bayer, Boehringer Ingelheim, GlaxoSmithKline, Resverlogix, Teikoku, New Amsterdam and Novo Nordisk; and is a director of Global Clinical Trial Partners. P.P. has received consulting fees from Boehringer Ingelheim, AstraZeneca, Vifor Pharma, Amgen, Servier, Novartis, Bayer, MSD, Pfizer, Cibiem, Impulse Dynamics, RenalGuard Solutions and BMS and honoraria from Boehringer Ingelheim, AstraZeneca, Vifor Pharma, Amgen, Servier, Novartis, Berlin Chemie, Bayer, Pfizer, Impulse Dynamics, RenalGuard Solutions, BMS and Abbott for lectures, presentations, speakers' bureaus, manuscript writing or educational events. Amina Rakisheva reports speaker honoraria fees from Bayer, Pfizer and Roche. F.R. received consulting fees from Air Liquide Santé International. G.S. reports grants and personal fees from Vifor, Boehringer Ingelheim, AstraZeneca, Novartis, Cytokinetics and Pharmacosmos; personal fees from Medtronic, Servier, TEVA, Edwards Lifesciences and INTAS; and grants from Boston Scientific, Merck and Bayer outside the submitted work. P.v.d.M. is supported by a grant from the European Research Council (ERC CoG 101045236, DISSECT‐HF). The UMCG, which employs PvdM, received consultancy fees and/or grants from Novartis, Pharmacosmos, Vifor Pharma, AstraZeneca, Pfizer, Pharma Nord, BridgeBio, Novo Nordisk, Daiichi Sankyo, Boehringer Ingelheim and Ionis. A.J.C. reports honoraria and/or lecture fees from AstraZeneca, Bayer, Boehringer Ingelheim, Edwards Lifesciences, Menarini, Novartis, Servier, Vifor, Abbott, Actimed, Arena, Cardiac Dimensions, Corvia, CVRx, Enopace, ESN Cleer, Faraday Pharmaceuticals, Impulse Dynamics, Respicardia and Viatris outside the submitted work. G.R. is supported by the Italian Ministry of Health (Ricerca Corrente). All other authors report no conflicts of interest.

## Supporting information


**Table S1.** Randomized controlled trials investigating the role of SGLT2 inhibitors in patients with acute or worsening HF and trials investigating the role of SGLT2 inhibitors on decongestion.
