# Supplementary material for: SGLT2 inhibitors for the prevention and treatment of heart failure: A scientific statement of the HFA and the HFAI
Source: ESC Heart Fail. 2025 Sep 19;12(6):3806–25. doi: 10.1002/ehf2.15408 (PMC12719827; doi:10.1002/ehf2.15408)
Supplement: Supplementary file 1 — Table S1. Randomized controlled trials investigating the role of SGLT2 inhibitors in patients with acute or worsening HF and trials investigating the role of SGLT2 inhibitors on decongestion. [file EHF2-12-3806-s001.docx]

**Supplementary Material**

**Supplementary Table 1. Randomized controlled trials investigating the role of SGLT2 inhibitors in patients with acute or worsening HF and trials investigating the role of SGLT2 inhibitors on decongestion.**

| **Study name and year** | **Drug comparison** | **Number of patients** | **Main eligibility criteria** | **Primary outcome** | **Effect of treatment on primary outcome** | **Additional findings** |
| --- | --- | --- | --- | --- | --- | --- |
| EMPA-RESPONSE-AHF, 2020^126^ | Empagliflozin 10 mg once daily vs placebo for 30 days | 80 patients | -Aged >18 years  -AHF, defined as all of the following: (i) dyspnoea at rest or with minimal exertion, (ii) signs of congestion (iii) BNP ≥350 pg/mL or NT-proBNP≥1400 pg/mL (if AF: BNP ≥500 pg/mL  or NT-proBNP ≥2000 pg/mL), and (iv) treated with loop diuretics at screening.  -eGFR ≥30 mL/min/1.73m2 between presentation and randomization | Change in VAS dyspnoea score, diuretic response (weight change per 40 mg  furosemide), change in NT-proBNP, and length of stay. | No difference was observed in VAS dyspnoea score, diuretic response, length of stay, or change in NT-proBNP. | -Empagliflozin reduced a combined endpoint of  in-hospital WHF, rehospitalization for HF or death at 60 days;  -Urinary output up until day 4 was significantly greater with empagliflozin  -No increase in the fractional excretion of sodium or 24-hour urinary sodium excretion at 4 or 30 days  -No change in body weight after 4 days.  - Increase in plasma osmolality with empagliflozin at 72  hours. |
| Tamaki et al, 2021^138^ | Empagliflozin 10 mg/daily  add-on or conventional glucose-lowering therapy within 96 h from admission. | 59 (terminated early due to COVID-19 pandemic) | -aged ≥20 years  -With T2DM  -Admitted for ADHF  -eGFR ≥15 mL/(min·1.73 m2) | Decongestion  as assessed based on NT-proBNP levels. | Lower Nt-proBNP at day 7 | -No changes in body weight at day 7  -Greater urine volume and greater urinary excretion of glucose and sodium during the first 24 hours  - Lower %ΔPV (but estimated inappropriately based on Strauss  formula)^  -Greater haemoconcentration at day 7 |
| SOLOIST-WHF, 2021 ^56^ | Sotagliflozinn vs placebo | 1,222 patients | -Aged 18 to 85 years  -Recent hospitalization due to WHF receiving iv diuretics  -With T2DM  -eGFR ≥ 30 ml/min/ 1.73 m2 | Total number of CV deaths and hospitalizations and urgent visits for HF (first and subsequent events). | HR, 0.67; 95% CI, 0.52 to 0.85 | -Benefits consistent across LVEF  - Starting sotagliflozin before discharge significantly decreased CV deaths and HF events through 30 and 90 days after discharge. |
| EMPAG-HF, 2022^128^ | Empagliflozin 25 mg daily or placebo within 12 hours of hospitalization  for 5 days | 60 | -Aged 18 to 85 years  -ADHF with signs of congestion  -BNP >100 pg/mL or NT-proBNP >300 pg/mL.  -eGFR ≥30 mL/min per 1.73 m² | Cumulative urine output over 5 days | -25% increase in cumulative  urine output over 5 days (median 10.8 versus 8.7 L in placebo, group difference estimation 2.2 L [95% CI, 8.4 to 3.6]).  -Increased diuretic efficiency compared with placebo (14.1 mL urine per milligram furosemide equivalent [95% CI, 0.6–27.7]) | -No significant differences in eGFR, nor in markers of renal injury  -More pronounced decrease in NTproBNP  -Trend towards reduction in body weight |
| EMPULSE, 2022 ^123,124,129^ | Empagliflozin 10 mg once daily vs placebo | 530 | - Aged ≥18 years  - Hospitalized due to AHF with signs of congestion (following stabilization between 24 h and 5 days after admission).*  - Any LVEF  - NT-proBNP ≥1600 pg/mL or BNP ≥400 pg/mL (if AF NT-proBNP ≥2400 pg/mL or BNP ≥600 pg/mL).  - Received at least 40mg of i.v. furosemide or equivalent. | Clinical benefit (a hierarchical composite of death from any cause, number of  HF events and time to first HF event, or a 5 point or greater difference in change from baseline in the KCCQ-TSS) at 90 days, as assessed using a win ratio. | Stratified win ratio, 1.36; 95% CI, 1.09–1.68. | Improvement in decongestion as assessed with weight loss (WL), WL adjusted for mean daily loop diuretic dose (WL-adjusted), change from baseline in NTproBNP levels, hemoconcentration, and clinical congestion score after 15, 30, and 90 days of treatment. |
| Yeoh SE et al., 2023^137^ | Dapagliflozin 10 mg once daily  vs metolazone 5–10 mg once daily for 3-days | 61 | -Adult patients  -hospitalized for WHF  -Any LVEF  -With diuretic resistance defined as insufficient decongestion (decrease in weight <1 kg or negative fluid balance <1 L) over the prior 24 h despite treatment with high dose IV loop diuretic (equivalent to ≥160 mg IV furosemide in 24 h) were eligible.  -BNP ≥ 100 pg/mL or NT-proBNP ≥ 400 pg/Ml  -persisting congestion  - eGFR ≥20 mL/min/1.73 m2. | Diuretic effect, assessed by change in weight (kg). | The mean (±SD)  decrease in weight at 96 h was 3.0 (2.5) kg with dapagliflozin compared to 3.6 (2.0) kg with metolazone [mean difference 0.65,  95% CI −0.12,1.41 kg; P = 0.11]. # | -Higher doses of loop diuretics needed with dapagliflozin  -Changes in pulmonary congestion and volume  assessment score were similar between treatments.  -Decreases in plasma sodium and potassium and increases in urea and  creatinine were smaller with dapagliflozin |
| DICTATE-AHF, 2024^135^ | Dapagliflozin 10 mg vs placebo | 240 | -Age ≥18 years  -AHF with congestion  -Anticipated or active use of IV loop diuretic  -eGFR ≥25 mL/min/1.73 m2 | Diuretic efficiency calculated as cumulative weight change divided by cumulative loop diuretic dose and expressed as kg/40 mg IV furosemide-equivalent | Odds ratio 0.65 (95% CI 0.41-1.01) | - Median cumulative loop diuretic dose was reduced with dapagliflozin vs. placebo: 560 vs. 800 mg, p = 0.006  - Dapagliflozin vs. placebo at 5 days: 24-hour natriuresis: 50 vs. 35 mmol/40 mg IV furosemide (p = 0.025)  24-hour diuresis: 634 vs. 403 mL/40 mg IV furosemide (p = 0.005) |
| DAPA-Shuttle1, 2024 | Dapagliflozin 10mg daily vs placebo for 4 weeks | 33 (29 provided accurate 24-hour urine collections) | -SGLT2i naive  -Patients with  chronic heart failure NYHA functional classes I/II  - Reduced LVEF | Change from baseline in urine osmolyte  Concentration. | Increase in urine glucose  excretion by 3.3 mmol/kg/d (95% CI: 2.51–4.04; P < 0.0001) within 48 hours (early) which persisted after 4 weeks (late;  2.7 mmol/kg/d [95% CI: 1.98–3.51]; P < 0.0001). | - Increased serum copeptin early and late leading to a reduction in  free water clearance and elevated urine concentrations  -No significant increase in urine  volume |

AF, atrial fibrillation; ADHF, acute decompensated heart failure; AHF, acute heart failure; BNP, -type natriuretic peptide; CI, confidence interval; CV, cardiovascular; eGFR, estimated glomerular filtration rate; HF, heart failure; HFH, hospitalisation for heart failure; HR, hazard ratio; IV, intravenous; KCCQ-TSS, Kansas City Cardiomyopathy Questionnaire Total Symptom Score; LVEF, left ventricular ejection fraction; NYHA, New York Heart Association; NT-proBNP, N-terminal pro B-type natriuretic peptide; OR, odds ratio; PV, plasma volume; SD, standard deviation; T2DM, type 2 diabetes mellitus; VAS, visual analogue scale; WHF, worsening heart failure; 6MWT, 6-minute walking test;

*****Patients are considered stabilized if they have: a systolic blood pressure ≥100mmHg and no symptoms of hypotension in the preceding 6 h; no increase in the intravenous (i.v.) diuretic dose for 6 h prior to randomization; no i.v. vasodilators including nitrates within the last 6 h, and no i.v. inotropic drugs for 24 h

#Patients in the dapagliflozin group received higher doses of furosemide (mean cumulative dose of furosemide at 96 h was 977 (±492) mg in the dapagliflozin group and 704 (±428) mg in the metolazone group)

^Tamaki et al. used the (%ΔPV) was calculated using the Strauss formula as follows: %ΔPV=([(hemoglobin1/hemoglobin2)×([100−hematocrit2]/[100−hematocrit1])]−1)×100 (%), where 1=baseline values and 2=subsequent values. PV is inappropriately estimated based on Strauss formula
